# Effect of Statins on Platelet Activation and Function: From Molecular Pathways to Clinical Effects

**DOI:** 10.1155/2021/6661847

**Published:** 2021-01-23

**Authors:** Antonio Nenna, Francesco Nappi, Mario Lusini, Umberto Maria Satriano, Davide Schilirò, Cristiano Spadaccio, Massimo Chello

**Affiliations:** ^1^Cardiovascular Surgery, Università Campus Bio-Medico di Roma, Rome, Italy; ^2^Cardiac Surgery, Centre Cardiologique du Nord de Saint Denis, Paris, France; ^3^Cardiac Surgery, Golden Jubilee National Hospital, Glasgow, UK

## Abstract

**Purpose:**

Statins are a class of drugs widely used in clinical practice for their lipid-lowering and pleiotropic effects. In recent years, a correlation between statins and platelet function has been unveiled in the literature that might introduce new therapeutic indications for this class of drugs. This review is aimed at summarizing the mechanisms underlying statin-platelet interaction in the cardiologic scenario and building the basis for future in-depth studies.

**Methods:**

We conducted a literature search through PubMed, Embase, EBSCO, Cochrane Database of Systematic Reviews, and Web of Science from their inception to June 2020.

**Results:**

Many pathways could explain the interaction between statins and platelets, but the specific effect depends on the specific compound. Some could be mediated by enzymes that allow the entry of drugs into the cell (OATP2B1) and others by enzymes that mediate their activation (PLA2, MAPK, TAX2, PPARs, AKT, and COX-1), recruitment and adhesion (LOX-1, CD36, and CD40L), or apoptosis (BCL2). Statins also appear to have a synergistic effect with aspirin and low molecular weight heparins. Surprisingly, they seem to have an antagonistic effect with clopidogrel.

**Conclusion:**

There are many pathways potentially responsible for the interactions between statins and platelets. Their effect appears to be closely related, and each single effect can be barely measured. Also, the same compound might have complex downstream signaling with potentially opposite effects, i.e., beneficial or deleterious. The multiple clinical implications that can be derived as a result of this interaction, however, represent an excellent reason to develop future in-depth studies.

## 1. Introduction

Platelet activation, oxidative stress, and endothelial integrity play a fundamental role in chronic inflammatory diseases such as atherosclerosis, and antiplatelet drugs are currently recommended in the treatment of coronary artery disease (CAD) [1–4]. In this scenario, statins have been demonstrated to reduce the incidence of cardiovascular events, such as myocardial infarction, stroke, and cardiovascular death [2]. Via the inhibition of 3-hydroxy-3-methyl-glutaryl-coenzyme A (HMG-CoA) reductase in the synthesis of endogen cholesterol, in addition to their lipid-lowering effect, statins reduce the progression of atherosclerosis and cardiovascular risk via inhibitory effects on inflammation and platelet aggregation. These effects, known to be pleiotropic, include changes in platelet function and half-life, reduction of oxidative stress, and protection of endothelial integrity [5]. Various molecular mechanisms underlie their clinical benefits, such as the variation of the platelet response to adenosine diphosphate (ADP), collagen, and arachidonic acid (AA) and the interaction with pro- and anti-inflammatory and atherogenic mediators (IL-1*β*, IL-5, IL-6, IL-7, IL-8, IL-9, IL-10, IL-12, and IL-13; IFN-*γ*, IP-10, eotaxin, and sRAGE; and HO-1), endothelial markers (s-selectin, VEGF. and MCP-1), and platelet (P-selectin, sCD-40L[6, 7], RANTES, and PDGF-bb) and oxidative stress (8-OH-2′-deoxyguanosine) activators. Furthermore, the interruption of statin therapy is associated with a marked increase in platelet activity [8]. Increase in platelet activity after statin withdrawal and the parallel loss of endothelial protection are two crucial and independent mechanisms that might contribute to a hypercoagulable state and thrombus formation, according to Virchow's triad.

The widespread use of statins and the multiple clinical implications that can be hypothesized starting from basic science studies should be carefully investigated in the cardiologic scenario. It appears crucial to summarize all the recent evidences on this topic with the aim of portraying the literary landscape and building the basis for future in-depth studies. This review is aimed at summarizing the main evidences available on the interaction between statins and platelet activity, with particular regard to the cardiovascular field.

## 2. Methods

For this narrative review, we evaluated all controlled randomized trials and retrospective studies investigating the effects of statin on platelet function. We conducted a literature search through PubMed, Embase, EBSCO, Cochrane Database of Systematic Reviews, and Web of Science from their inception to June 2020, using the following search keywords in various combinations: (“Hydroxymethylglutaryl-CoA Reductase Inhibitors”[Mesh] OR statin OR statins OR atorvastatin OR rosuvastatin OR lovastatin OR pravastatin OR simvastatin OR ∗statin) AND (“Blood Platelets”[Mesh] OR “Thrombocytopenia”[Mesh] OR “Thrombocytosis”[Mesh]). We also reviewed references of previous systematic reviews, meta-analysis, and abstracts from major congresses. Two investigators independently reviewed the studies to determine their eligibility and independently extracted all the relevant outcomes of interest.

## 3. Results

### 3.1. Molecular Pathways

Numerous studies have investigated the relationship between statin therapy and platelet activity and established the main mechanisms responsible for their biological effects (Table 1).

The anion-transporter polypeptide OATP2B1, expressed on the platelet membrane, could play a crucial role in this interaction. Niessen et al. and Jedlitschky et al. have shown that statin uptake into platelets may be mediated by this macromolecule, which has a high affinity for atorvastatin. The level of its expression could justify the variable effect of statins on platelet inhibition [9, 10].

Zhao et al. demonstrated that atorvastatin is able to inhibit aggregation in platelet extracts previously treated with ADP (10 mmol/L), arachidonic acid (0.5 mmol/L), collagen (2 mg/mL), and heparin (1 mg/mL) at moderate (300 × 10^9^/L) and high (600 × 10^9^/L) concentrations [11]. These findings were also confirmed by Akyüz et al. using platelet volume (MPV) as a marker of platelet activity in patients undergoing rosuvastatin therapy [12]. However, the use of MPV as a direct marker of platelet activation is not a gold standard and is not widely accepted. Moreover, changes of MPV could derive from an alteration of platelet turnover and megakaryopoiesis: a reduced platelet production leading to a lower quote of circulant immature or reticulated platelets could explain these findings. Therefore, future tailored studies are warranted to support those findings.

These effects could be explained by the inhibition of platelet phospholipase A2 (PLA2) phosphorylation and the MAP kinase pathway with consequent reduction of intracytoplasmic calcium release and dose-dependent inhibition of collagen-induced synthesis of thromboxane A2 (TXA2) [13–17]. Moreover, recent studies suggest possible interaction between statins and nuclear transcription factors such as peroxisome proliferator-activated receptors (PPARs) involved in the modulation of the C-*α* platelet protein kinase [11]. Notably, simvastatin therapy has been reported to modulate the PPAR alpha and PPAR gamma pathway [18–20]. These macromolecules, in turn, mediate the activity of multiple other cellular mediators such as AKT, cAMP, ERK, p38, and MAPK as well as the concentration of cytoplasmic Ca, leading altogether to a significant attenuation of platelet activity [18–20].

Other studies have shown that the antiplatelet effects of statins could be related to the upregulation of nitric oxide synthetase (NOS) and the downregulation of cyclooxygenase-1 (COX-1) activation [21–23].

Another hypothesis was advanced by Lee et al. who identified as the main actors of statin-induced platelet inhibition both the upregulation of heme oxygenase-1 (HO-1, an anti-inflammatory, antioxidant, and cytoprotective enzyme [24]) and the reduction of the type 1 collagen expression. Rosuvastatin-loaded nanofibers in biodegradable stents showed a significant reduction of inflammation and an evident decrease in migration and intrastent adhesion of platelets. Importantly, the combination of the two effects (anti-inflammatory and antiplatelet) also allowed an improved reendothelialization of the stent and a decrease in neointimal formation of the injured artery [25].

On the other hand, the downregulation of specific ox-LDL receptors such as CD36 and LOX-1 has a biological role in the increase in platelet activation and adhesion [17, 26–29].

Furthermore, Pignatelli et al. reported that statins may reduce platelet recruitment by inhibiting nicotinamide adenine dinucleotide phosphate (NADPH) oxidase activation and in particular its subunit with NOX-2 catalytic activity. The activity of this enzyme would in fact be fundamental for the production of isoprostanes, responsible for platelet activation. Thus, its downregulation would lead to a significant attenuation in both platelet recruitment and activity but also to a decreased production of proinflammatory factors such as CD40L and reactive oxygen species (ROS). Conversely, levels of anti-inflammatory factors such as nitric oxide (NO) would increase following statin-mediated platelet NADPH oxidase inhibition [23, 30–32] [16, 20, 33]. The evidence of a possible antiplatelet effect of statins attributable to the downregulation of the platelet CD40L pathway was also confirmed by an in vitro study of Sanguigni et al. [34] using hypercholesterolemic patients (randomized to atorvastatin or diet) and healthy volunteers. These authors showed that atorvastatin can reduce the in vitro expression of CD40L and therefore downgrade platelet activation through the inhibition of clotting activation by CD40L-stimulated monocytes. This mechanism may be particularly relevant in patients with hypercholesterolemia, characterized by an overexpression of CD40L [34] [35]. However, the absence of functional and in vivo experimental models partially limits the generalization of those findings.

Statin therapy has been reported to increase NO basal levels and activity through an upregulation of endothelial NO synthase (eNOS). The consequent decrease in platelet function translates to a prolongation of patient bleeding time. The NO pathway could also modulate intraplatelet calcium levels, determining a synergistic inhibitory effect to platelet activation [36].

Another possible mediator of the decrease in platelet activity is the platelet thrombin receptor PAR-1, which is one of the strongest activation pathways in platelets [37]. Serebruany et al. observed a significant reduction in the activity and concentration of this macromolecule in patients treated with statins. This would entail not only a reduction in platelet-aggregating capacity but also a decrease in the effectiveness of the coagulation cascade [37]. In detail, Serebruany et al. included patients with metabolic syndrome without antiplatelet therapy, receiving atorvastatin, fluvastatin, lovastatin, pravastatin, rosuvastatin, or simvastatin or no statin for 6 weeks. The PAR-1 thrombin receptor was investigated considering both intact (SPAN12) and cleaved (WEDE15) forms during and at the end of treatment. The statin-mediated inhibition was stronger after 4 weeks with a small rebound at complete treatment, with different temporal patterns of inhibition between intact and cleaved forms. This study was a milestone in providing a mechanism for the pleiotropic effect of statins that can lead to early clinical benefits.

Statins may also affect platelet energy metabolism. Vevera et al. reported a reduction of mitochondrial platelet respiration in a rat model treated with rosuvastatin and atorvastatin. However, the same effect was not found in human models by the same investigators [38].

In contrast with these observations in the study by Panes et al., atorvastatin did not result in reduced levels of tissue factor (TF) and tissue factor-dependent procoagulant activity (TF-PCA). Furthermore, atorvastatin did not decrease cholesterol levels in the platelet membrane. Rosuvastatin therapy would instead be able to achieve these effects, ultimately leading to a downregulation of platelet activity [39].

CD62 ligand (CD62L, L-selectin) is another target of statins and mediates their antiplatelet effects. Labiós et al. have shown a reduction in the expression of this ligand in patients undergoing statin therapy. In a cytofluorometry study, these authors demonstrated a decrease in platelet activity as well as an attenuation of patients' prothrombotic profile [40, 41]. Furthermore, Pontremoli et al. have speculated that the DKK-1 pathway might be influenced by statin treatment. The concentration of these proteins, members of the Dickkopf family, would be decreased after statin administration, resulting in diminished platelet recruitment and activity [42].

Suades et al. highlighted a statin-induced reduction in circulating microparticles (cMPS), which physiologically work as mediators for the proinflammatory activity of numerous cells, including platelets. A reduction in circulating cMPS levels, in particular those related to platelets, has been shown in patients undergoing statin therapy [43]. Moreover, a reduction of inflammatory markers such as TF, P-selectin, CD14, and GPIIIa on the cMPS surface has been shown after therapy with atorvastatin [44–46].

Another molecule potentially involved in statin-related reduction of platelet activity is the protein regulated by glucose 78 (GRP78). This macromolecule can be found both in the cytoplasm and in the platelet membrane; besides having a role of chaperon protein, it would increase the sensitivity of the platelets to various activating factors. Rosuvastatin therapy has been demonstrated to reduce the translocation of this protein on the platelet membrane, its activation, and subsequent platelet aggregation [47].

Statin therapy also appears to modify, through mechanisms not yet fully clarified, the expression of the miRNA networks. In their study, Li et al. have shown the possible correlation between the miRNA-33 upregulation and an increase in plaque stability in hypercholesterolemic patients. In particular, they showed a reduction in multiple inflammatory mediators, with a consequent decrease in platelet adhesion and activation [48].

Another theory hypothesizes that statin therapy may induce an increase in platelet apoptosis rather than a reduction in their functionality. In this regard, Zhao et al. described how BCL2 modulation, caspase, and TNF pathways determine an overall increase in the platelet proapoptotic profile [5].

To conclude, from a comprehensive analysis of the literature, it appears that an interaction exists between statins and platelet activity. This is partly mediated by the effect produced by these drugs on inflammatory agents such as NO, HO-1, ROS, and NOX-2 which are potent platelet activators as well. Furthermore, the effect shown by statins on enzymes such as PLA2, TXA2, or eNOS could further explain their influence on platelet activity. The variable impact that different statins may have on the pathways mentioned before could be related to their different molecular composition. In particular, the lipophilic or hydrophilic nature of the molecule appears to have a significant role. The molecular structure may justify the ability to selectively interact with some ligands, bind with specific receptors, or facilitate the entry of the drug into its target cell.

### 3.2. Synergistic Effects with Other Drugs

Statins may also have synergistic effects with other drugs. Luzak et al. have shown that statin therapy increased the effect of the acetylsalicylic acid (ASA) on platelets of hypercholesterolemic patients. Indeed, the acetylation of platelet proteins induced by ASA is increased in patients undergoing concomitant treatment with statins. This could be explained by a qualitative modification of the platelet cell membrane justifying the higher sensitivity to ASA treatment [49]. Furthermore, statin therapy might increase sensitivity to ASA by decreasing any effects of tolerance to this drug in patients with CAD [7, 29, 50].The underexpression of the GPIIb-IIIa protein on the platelet surface found in patients treated with simvastatin or pravastatin and ASA is another aspect of this synergistic effect [29]. Moreover, in patients treated with pravastatin and ASA, a reduction in lecithin-like oxidized LDL receptor-1 (LOX-1) expression has been observed [28].

On the other hand, statins, in particular atorvastatin, could have an antagonistic effect towards clopidogrel. Atorvastatin would be able to inhibit the isoenzyme necessary to metabolize clopidogrel in its active form, thus potentially reducing its antiaggregating effect [51].

Statins may also increase the anticoagulation activity of low molecular weight heparins (LMWHs). In patients treated with both drugs, Zimmer et al. documented an increase in clotting time. The cause could be a possible effect of statins on the coagulation cascade and/or the addition of an antiplatelet effect [52].

The interaction between statins and other drugs could have significant clinical implications. Patients taking statins regularly often receive polydrug therapies. The association with ASA is, among all, the most noticeable, and their synergistic effect warrants future studies. On the other hand, the possible antagonistic effect shown with clopidogrel suggests the possibility of investigating the potential implications of the use of the two drugs in several conditions such as acute coronary syndromes.

## 4. Discussion

### 4.1. Clinical Implications

On the basis of the experimental evidence of an interaction among statins and platelet function, several clinical studies have been conducted to evaluate potential therapeutic benefits of statins in conditions characterized by an enhanced thrombotic state, independently of their lipid-lowering activity (Table 2).

Kong et al. investigated the possible effects of using statins in corticosteroid-resistant immune thrombocytopenia (ITP). In this condition, treatment with atorvastatin was associated with a quantitative and functional improvement of endothelial progenitor cells resident in the bone marrow. A downregulation of MAPK p38 and an upregulation of the Akt pathway have been suggested among the possible mechanisms. Moreover, atorvastatin partially contributed to the repair of damaged endothelial progenitors via an increase in megakaryocytopoiesis [53].

Atherosclerosis is another disease in which the antiplatelet effects of statin therapy have been extensively investigated. In a study by Konishi et al. on patients with carotid atherosclerosis, treatment with statins resulted in reduced thrombotic complications such as plaque rupture and embolic/thrombotic events, intraplaque hemorrhage, or aneurysmal degeneration. They also performed a histological analysis of vessel samples from patients treated with statins and found a decreased activation and migration of inflammatory cells and platelets, as well as a reduction of intraplaque angiogenetic phenomena [54].

In patients with acute coronary syndromes, early therapy with high-dose statins significantly reduced the interaction between platelets and circulating leukocytes, thus reducing the proinflammatory and prothrombotic profile of patients affected by this pathology [35, 37, 55].

Although a pharmacodynamic interaction between clopidogrel and cytochrome CYP3A4-metabolized statins has been described, the ACHIDO (Atorvastatin and Clopidogrel HIgh DOse in stable patients with residual high platelet activity) study [56] found that treatment with 80 mg of atorvastatin was associated with optimal pharmacodynamic response to clopidogrel (defined as P2Y12 reaction units (PRU) < 235 by the VerifyNow P2Y12 assay, OR 3.8 (*P* = 0.011)); also, statin effect size was greater than genetic variants (CYP2C19∗2 loss-of-function allele, odds ratio 2.9, *P* = 0.043). Therefore, in stable CAD patients undergoing percutaneous coronary intervention (PCI), the addition of high-dose atorvastatin significantly improves the pharmacodynamic effects of high-dose clopidogrel and is associated with lower rates of drug resistance. The reduction of endothelial inflammatory response may partially explain this protective effect of statins, as shown in the ARMYDA study (Atorvastatin for Reduction of MYocardial Damage during Angioplasty) [57] [58], which found that atorvastatin treatment was associated with attenuated increase in ICAM-1 and E-selectin levels after PCI.

The interaction between platelets and endothelial cells could also be affected by statin therapy. On mouse models affected by arthritic pathology, a reduction between platelet-to-endothelial cell adhesion has been shown in animals receiving simvastatin therapy. This effect might lead to a reduction in the proinflammatory and prothrombotic platelet potential [59]. Statin therapy, on the other hand, did not seem to affect the pool of immature platelet cells, keeping their regeneration unaffected [60].

Another class of pathologies in which the effect of statin has been investigated is myeloproliferative pathologies (polycythemia vera, essential thrombocythemia, and idiopathic myelofibrosis). In fact, in these patients, statin therapy could reduce the risk of thromboembolic phenomena given their anti-inflammatory, endothelial protector, and platelet inhibition effects. Additionally, statins have shown a proapoptotic effect on leukemic cells as well as an antiangiogenetic effect, allowing also the speculation of a cytoreductive action [61].

### 4.2. Statins and Bleeding

Statin therapy appears to be associated with reduced postoperative bleeding following major surgery, although the underlying molecular mechanisms are not fully understood. Nenna et al. showed a reduction in postoperative bleeding in patients on statins undergoing aortic valve replacement and coronary artery bypass surgery [62, 63]. Despite the fact that a potential attenuation of the inflammatory response related to cardiopulmonary bypass might play a role, the relation between bleeding and microvascular permeability requires further demonstration.

Falcone et al. showed an inverse correlation between the use of statins and intracranial cerebral hemorrhage [64]. This relationship is also confirmed by Quinn et al. that reported a reduction in risk of intracranial hemorrhage in patients with ischemic stroke treated with statins [65]. Early statin therapy following cerebral hemorrhage has also been demonstrated to be protective against the onset of new episodes [66].

Moreover, Atar et al. reported an inverse relationship between gastrointestinal bleeding and platelet therapy in patients hospitalized for acute coronary syndrome [67].

On this ground, a relation between statin and bleeding reduction is plausible [67]. Notably, statins may be useful in reducing bleeding regardless of its cause. While in the report by Nenna et al., the anti-inflammatory action of statins seems to play a decisive role in reducing hemorrhagic complications, in the study by Atar et al., statin effect seems independent of patients' inflammatory profile.

Considering both the adverse outcomes and the increase in health care costs necessary to deal with hemorrhagic complications (transfusions, etc.), further investigations on the potential mechanisms underlying statin effects in bleeding complications are warranted.

### 4.3. Limitations

Methodological limitations and biases are extremely common in the scientific literature evaluating the effect of statins and platelets. The high number of confounding factors remains a daunting issue, as in vitro studies usually focus on one specific pathway and this barely represents the complexity of a living system. Also, some studies might have not been included in this literature search due to different keywords or deficit in database indexing.

## 5. Conclusion

**Figure 1:**
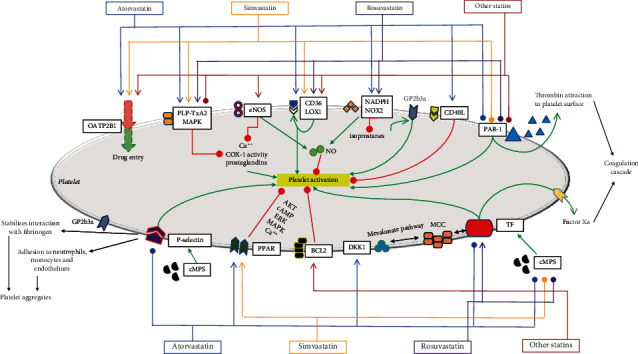
Detailed summary of platelet-statin interactions. A green (sharp point) arrow indicates a stimulatory pathway. A red (round point) arrow indicates an inhibitory pathway. Different statins are marked with different colors. See text for abbreviations. “Other statins” indicate compounds different from atorvastatin, simvastatin, or rosuvastatin that have a minor amount of supporting literature for each compound.

**Figure 2:**
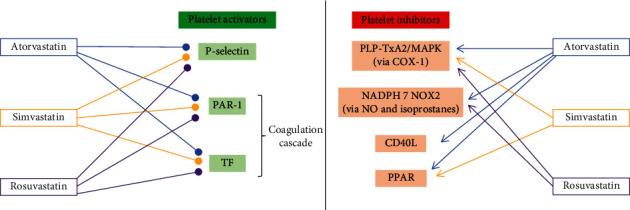
Statin categorization according to their inhibitory or activating effect. A sharp point arrow indicates an activating effect. A round point arrow indicates an inhibitory effect. See text for abbreviations.

This narrative review describes the interactions between statins and platelets (Figures 1and 2). The primary prevention of cardiovascular disease might be revised based on the available basic science studies (Table 2). Surely, further studies to identify the molecular mechanisms and confirm the clinical effectiveness are warranted. A plethora of molecular pathways are potentially involved, and a comprehensive understanding of their interaction remains a goal of the current literature. In conclusion, the interactions between tissue factor, CD40L, and P-selectin appear to be the most promising for further research; this highlights the importance of statin as an endothelial modulator and outlines this mechanism as a factor in the prevention of thrombotic events. Statin use might reduce platelet activation via inhibition of P-selectin, activation of PPAR, activation of BCL2, activation of CD40L, or upstream blockage of tissue factor, with consequent impairment of coagulation cascade activation. Moreover, platelet activation can be hampered by eNOS- or NADPH-dependent pathways possibly via their effects on NO bioavailability. There are many pathways potentially responsible for the interactions between statins and platelets. Their effect appears to be closely related, and each single effect can be barely measured. Also, the same compound might have complex downstream signaling with potentially opposite effects, i.e., beneficial or deleterious; multiple pathways cooperate, and a synergic effect is most likely to determine the overall strength of the interaction. Nonetheless, the possibility of identifying a “critical” pathway merits further investigation. The extensive clinical use of statins fully justifies such a scientific effort given the enormous implications that new evidences on statin-platelet interaction may have.

## Figures and Tables

**Table 1:** Statin-specific molecular targets and their downstream effectors.

Molecular target	Downstream effect	Statin effect	References
OATP2B1	Drug entry into the platelet	Atorvastatin (act) Simvastatin (act) Pravastatin (act)	[9]

Phospholipase A2-thromboxane A2 (TXA2), MAP kinase	↓ Ca inside the platelets ↓ COX-1 activity ↓ quantity and activity of prostaglandins	Pravastatin (inact) Simvastatin (act) Atorvastatin (act) Rosuvastatin (act)	[13] [14] [15] [16] [17]

eNOS (NO synthase)	↑ NO ↓ Ca inside the platelets	Pravastatin (act)	[36]

HO-1	Antioxidant effect	Rosuvastatin (act)	[24]

CD36, LOX1 (ox-LDL receptors)	Modulation of the quantity and quality of these two receptors, both strong platelet activators	Atorvastatin (act) Pravastatin (act) Simvastatin (act) Rosuvastatin (act)	[26] [27] [28] [29] [17]

NADPH-NOX-2	↓ isoprostanes, family of chemically stable eicosanoids that contribute to propagation of platelet activation via upregulation of the glycoprotein IIb/IIIa (GpIIb/IIIa) ↓ ROS ↑ NO ↓ PKC phosphorylation and p47phox translocation	Rosuvastatin (act) Atorvastatin (act)	[30] [23] [31] [32] [16] [33] [20]

CD40L	↓ proinflammatory and prothrombotic activity, including increased expression of matrix metalloproteinases and chemokines	Atorvastatin (act)	[34]

PAR-1 (protease-activated receptor-1)	↓ PAR-1, responsible for attracting thrombin to the platelet surface, serving as a modulator between platelet activation and thrombin formation and its shredding from the cell surface	Atorvastatin (act) Fluvastatin (act) Lovastatin (act) Pravastatin (act) Rosuvastatin (act) Simvastatin (act)	[37]

Mitochondrial respiration enzymes (complex I-linked respiration)	Unknown	Simvastatin (act)	[38]

TF, TF-PCA, membrane cholesterol content	GPIba-mediated activation of platelet TF triggers the generation of FXa Platelet TF has the capacity to initiate the clotting process Membrane cholesterol content plays important roles in platelet activation and calcium signaling	Rosuvastatin (act) Atorvastatin (inact)	[39]

CD62 (P-selectin)	↓ P-selectin regulates adhesion of activated platelets to neutrophils and monocytes and also to the endothelium and stabilizes the initial GPIIb/IIIa-fibrinogen interaction, allowing the formation of large, stable platelet aggregates	Atorvastatin (inact)	[40]

DKK-1	↓ mevalonate pathway	Atorvastatin (act)	[42]

PPAR alfa and PPAR gamma	↓ AKT ↑ cAMP ↓ ERK ↓ p38 ↓ MAPK ↓ Ca cytosol ↓ protein kinase C	Simvastatin (act) Atorvastatin (act)	[18] [11]

Circulating microparticles (cMPS)	TF (tissue factor) P-selectin CD14 GPIIIa	Atorvastatin (inact) Pravastatin (inact) Simvastatin (inact) Rosuvastatin (inact) Lovastatin(inact)	[46] [43] [44] [45]

GRP78	Chaperon protein	Rosuvastatin (act)	[47]
miRNA	miR-155 expression through interfering with the mevalonate-geranylgeranyl-pyrophosphate-RhoA signaling pathway and then increasing endothelial NO synthase expression and endothelium-dependent vasodilation	Atorvastatin (act) Simvastatin (act)	[48]

BCL2-caspase, TNF	↑ apoptosis	Lovastatin (act)	[2]

Statin effect on the molecular pathway: activates (act)/inactivates (inact). See text for abbreviations.

**Table 2:** Potential new clinical indications for statins in the primary prevention of cardiovascular disease (adapted from 2019 ESC/EAS Guidelines for the management of dyslipidemias).

Total cardiovascular risk (%)	Untreated LDL level (pretreatment)					
	<55 mg/dL	55-70 mg/dL	70-100 mg/dL	100-116 mg/dL	116-190 mg/dL	>190 mg/dL
<1% low risk	Lifestyle interventions	Lifestyle interventions	Lifestyle interventions	**Statin might be considered**	Statin might be considered	Statin indicated
1-5% moderate risk	Lifestyle interventions	Lifestyle interventions	**Statin might be considered**	Statin might be considered	Statin might be considered	Statin indicated
5-10% high risk	Lifestyle interventions	**Statin might be considered**	Statin might be considered	Statin indicated	Statin indicated	Statin indicated
>10% very high risk	**Statin might be considered**	Statin might be considered	Statin indicated	Statin indicated	Statin indicated	Statin indicated

Adapted from 2019 ESC/EAS Guidelines for the management of dyslipidemias (doi: 10.1093/eurheartj/ehz455). Bold indicates differences from guideline recommendations.

## Data Availability

No data were used to support this study.

## References

[1] Davì G., Patrono C. (2007). Platelet activation and atherothrombosis. *The New England Journal of Medicine*.

[2] Gallone G., Baldetti L., Pagnesi M. (2018). Medical therapy for long-term prevention of atherothrombosis following an acute coronary syndrome. *Journal of the American College of Cardiology*.

[3] Lordan R., Tsoupras A., Zabetakis I. (2020). Platelet activation and prothrombotic mediators at the nexus of inflammation and atherosclerosis: potential role of antiplatelet agents. *Blood Reviews*.

[4] Spadaccio C., Antoniades C., Nenna A. (2019). Preventing treatment failures in coronary artery disease: what can we learn from the biology of in-stent restenosis, vein graft failure, and internal thoracic arteries?. *Cardiovascular Research*.

[5] Zhao Q., Li M., Chen M. (2016). Lovastatin induces platelet apoptosis. *Environmental Toxicology and Pharmacology*.

[6] Filozof C., Gómez-Garre D., Reinares L. (2008). Relationship between plasma levels of soluble CD40L and insulin sensitivity and insulin secretion status in non-diabetic dyslipidemic patients. *Diabetes Research and Clinical Practice.*.

[7] Barale C., Frascaroli C., Senkeev R., Cavalot F., Russo I. (2018). Simvastatin effects on inflammation and platelet activation markers in hypercholesterolemia. *BioMed Research International.*.

[8] Puccetti L., Pasqui A. L., Pastorelli M. (2003). Platelet hyperactivity after statin treatment discontinuation. *Thrombosis and Haemostasis.*.

[9] Niessen J., Jedlitschky G., Grube M. (2009). Human platelets express Organic Anion-Transporting Peptide 2B1, an uptake transporter for atorvastatin. *Drug Metabolism and Disposition*.

[10] Jedlitschky G., Greinacher A., Kroemer H. K. (2012). Transporters in human platelets: physiologic function and impact for pharmacotherapy. *Blood*.

[11] Zhao L., Liu D., Liu B., Hu H., Cui W. (2017). Effects of atorvastatin on ADP-, arachidonic acid-, collagen-, and epinephrine-induced platelet aggregation. *Journal of International Medical Research.*.

[12] Akyüz A., Akkoyun D. Ç., Değirmenci H., Oran M. (2015). Rosuvastatin decreases mean platelet volume in patients with diabetes mellitus. *Angiology*.

[13] Moscardó A., Vallés J., Latorre A., Madrid I., Santos M. T. (2013). Reduction of platelet cytosolic phospholipase A2 activity by atorvastatin and simvastatin: biochemical regulatory mechanisms. *Thrombosis Research.*.

[14] Liao J. K. (2005). Effects of statins on 3-hydroxy-3-methylglutaryl coenzyme a reductase inhibition beyond low-density lipoprotein cholesterol. *American Journal of Cardiology*.

[15] Hamilton P. K., Hughes S. M. T., Plumb R. D. (2010). Statins have beneficial effects on platelet free radical activity and intracellular distribution of GTPases in hyperlipidaemia. *Clinical Science.*.

[16] Violi F., Calvieri C., Ferro D., Pignatelli P. (2013). Statins as antithrombotic drugs. *Circulation*.

[17] Puccetti L., Santilli F., Pasqui A. L. (2011). Effects of atorvastatin and rosuvastatin on thromboxane-dependent platelet activation and oxidative stress in hypercholesterolemia. *Atherosclerosis*.

[18] Du H., Hu H., Zheng H., Hao J., Yang J., Cui W. (2014). Effects of peroxisome proliferator-activated receptor *γ* in simvastatin antiplatelet activity: influences on cAMP and mitogen-activated protein kinases. *Thrombosis Research.*.

[19] Ali F. Y., Armstrong P. C. J., Dhanji A. R. A. (2009). Antiplatelet actions of statins and fibrates are mediated by PPARs. *Arteriosclerosis, Thrombosis, and Vascular Biology.*.

[20] Oesterle A., Laufs U., Liao J. K. (2017). Pleiotropic effects of statins on the cardiovascular system. *Circulation Research.*.

[21] Gocmen A. Y., Ocak G. A., Ozbilim G., Delibas N., Gumuslu S. (2013). Effect of atorvastatin on atherosclerotic plaque formation and platelet activation in hypercholesterolemic rats. *Canadian Journal of Physiology and Pharmacology.*.

[22] Laufs U., Gertz K., Huang P. (2000). Atorvastatin upregulates type III nitric oxide synthase in thrombocytes, decreases platelet activation, and protects from cerebral ischemia in normocholesterolemic mice. *Stroke*.

[23] Violi F., Carnevale R., Pastori D., Pignatelli P. (2014). Antioxidant and antiplatelet effects of atorvastatin by Nox2 inhibition. *Trends in Cardiovascular Medicine.*.

[24] Loboda A., Jazwa A., Grochot-Przeczek A. (2008). Heme oxygenase-1 and the vascular bed: from molecular mechanisms to therapeutic opportunities. *Antioxidants and Redox Signaling.*.

[25] Lee C. H., Chang S. H., Lin Y. H. (2014). Acceleration of re-endothelialization and inhibition of neointimal formation using hybrid biodegradable nanofibrous rosuvastatin-loaded stents. *Biomaterials*.

[26] Puccetti L., Pasqui A., Pastorelli M. (2005). 3’UTR/T polymorphism of lectin-like oxidized low-density lipoprotein receptor-1 (LOX-1) is associated with modified anti-platelet activity of atorvastatin in hypercholesterolemic subjects. *Atherosclerosis*.

[27] Phillip Owens A., Mackman N. (2014). The antithrombotic effects of statins. *Annual Review of Medicine*.

[28] Marwali M. R., Hu C. P., Mohandas B. (2007). Modulation of ADP-induced platelet activation by aspirin and pravastatin: role of lectin-like oxidized low-density lipoprotein receptor-1, nitric oxide, oxidative stress, and inside-out integrin signaling. *Journal of Pharmacology and Experimental Therapeutics.*.

[29] Luzak B., Rywaniak J., Stanczyk L., Watala C. (2012). Pravastatin and simvastatin improves acetylsalicylic acid-mediated in vitro blood platelet inhibition. *European Journal of Clinical Investigation.*.

[30] Pignatelli P., Carnevale R., Di Santo S. (2012). Rosuvastatin reduces platelet recruitment by inhibiting NADPH oxidase activation. *Biochemical Pharmacology*.

[31] Pignatelli P., Sanguigni V., Lenti L. (2007). Oxidative stress-mediated platelet CD40 ligand upregulation in patients with hypercholesterolemia: effect of atorvastatin. *Journal of Thrombosis and Haemostasis*.

[32] Ni R., Peleg T., Gross P. L. (2012). Atorvastatin delays murine platelet activation in vivo even in the absence of endothelial NO synthase. *Arteriosclerosis, Thrombosis, and Vascular Biology.*.

[33] Roberto C., Pasquale P., Serena D. S. (2010). Atorvastatin inhibits oxidative stress via adiponectin-mediated NADPH oxidase down-regulation in hypercholesterolemic patients. *Atherosclerosis*.

[34] Sanguigni V., Pignatelli P., Lenti L. (2005). Short-term treatment with atorvastatin reduces platelet CD40 ligand and thrombin generation in hypercholesterolemic patients. *Circulation*.

[35] Sexton T., Wallace E., Smyth S. (2016). Anti-thrombotic effects of statins in acute coronary syndromes: at the intersection of thrombosis, inflammation, and platelet-leukocyte interactions. *Current Cardiology Reviews.*.

[36] Yemisci M., Ay H., Kocaefe Ç. (2008). Statin potentiates human platelet eNOS activity without enhancing eNOS mRNA and protein levels. *Cerebrovascular Diseases*.

[37] Serebruany V. L., Miller M., Pokov A. N. (2006). Effect of statins on platelet PAR-1 thrombin receptor in patients with the metabolic syndrome (from the PAR-1 inhibition by statins [PARIS] study). *American Journal of Cardiology*.

[38] Vevera J., Fišar Z., Nekovářová T. (2016). Statin-induced changes in mitochondrial respiration in blood platelets in rats and human with dyslipidemia. *Physiological Research*.

[39] Panes O., González C., Hidalgo P. (2017). Platelet tissue factor activity and membrane cholesterol are increased in hypercholesterolemia and normalized by rosuvastatin, but not by atorvastatin. *Atherosclerosis*.

[40] Labiós M., Martínez M., Gabriel F., Guiral V., Martínez E., Aznar J. (2005). Effect of atorvastatin upon platelet activation in hypercholesterolemia, evaluated by flow cymetry. *Thrombosis Research.*.

[41] Sadowitz B., Maier K. G., Gahtan V. (2010). Basic science review: statin therapy-part I: the pleiotropic effects of statins in cardiovascular disease. *Vascular and Endovascular Surgery.*.

[42] Pontremoli M., Brioschi M., Baetta R., Ghilardi S., Banfi C. (2018). Identification of DKK-1 as a novel mediator of statin effects in human endothelial cells. *Scientific Reports*.

[43] Suades R., Padró T., Alonso R., Mata P., Badimon L. (2017). Lipid-lowering therapy with statins reduces microparticle shedding from endothelium, platelets and inflammatory cells. *Thrombosis and Haemostasis*.

[44] Montoro-García S., Lip G. Y. H., Shantsila E. (2011). Atorvastatin and its collateral effects on microparticles. *Thrombosis and Haemostasis.*.

[45] Mobarrez F., He S., Bröijersen A. (2011). Atorvastatin reduces thrombin generation and expression of tissue factor, p-selectin and GPIIIa on platelet-derived microparticles in patients with peripheral arterial occlusive disease. *Thrombosis and Haemostasis*.

[46] Sommeijer D. W., Joop K., Leyte A., Reitsma P. H., Ten Cate H. (2005). Pravastatin reduces fibrinogen receptor gpIIIa on platelet-derived microparticles in patients with type 2 diabetes. *Journal of Thrombosis and Haemostasis*.

[47] Molins B., Peña E., Padro T., Casani L., Mendieta C., Badimon L. (2010). Glucose-regulated protein 78 and platelet deposition. *Arteriosclerosis, Thrombosis, and Vascular Biology.*.

[48] Li S., Cao C., Chen H. (2017). Atheroprotective effects of statins in patients with unstable angina by regulating the blood-borne microRNA network. *Molecular Medicine Reports.*.

[49] Luzak B., Boncler M., Rywaniak J. (2011). The effect of a platelet cholesterol modulation on the acetylsalicylic acid-mediated blood platelet inhibition in hypercholesterolemic patients. *European Journal of Pharmacology.*.

[50] Tirnaksiz E., Pamukcu B., Oflaz H., Nisanci Y. (2009). Effect of high dose statin therapy on platelet function; statins reduce aspirin resistant platelet aggregation in patients with coronary heart disease. *Journal of Thrombosis and Thrombolysis.*.

[51] Tentzeris I., Siller-Matula J., Farhan S., Jarai R., Wojta J., Huber K. (2011). Platelet function variability and non-genetic causes. *Thrombosis and Haemostasis*.

[52] Zimmer J. E., Spillert C. R., Puppala S., Zamecki K., Bhatt B. A., Arora R. R. (2004). Pravastatin potentiates the anticoagulant effects of low molecular weight heparin. *Thrombosis Research.*.

[53] Kong Y., Cao X. N., Zhang X. H. (2018). Atorvastatin enhances bone marrow endothelial cell function in corticosteroid-resistant immune thrombocytopenia patients. *Blood*.

[54] Konishi T., Funayama N., Yamamoto T. (2018). Stabilization of symptomatic carotid atherosclerotic plaques by statins: a clinico-pathological analysis. *Heart and vessels.*.

[55] Sexton T. R., Wallace E. L., Macaulay T. E. (2015). The effect of rosuvastatin on platelet-leukocyte interactions in the setting of acute coronary syndrome. *Journal of the American College of Cardiology.*.

[56] Leoncini M., Toso A., Maioli M. (2013). High-dose atorvastatin on the pharmacodynamic effects of double-dose clopidogrel in patients undergoing percutaneous coronary interventions: the ACHIDO (Atorvastatin and Clopidogrel HIgh DOse in stable patients with residual high platelet activity) study. *JACC: Cardiovascular Interventions.*.

[57] Patti G., Chello M., Gatto L. (2010). Short-term atorvastatin preload reduces levels of adhesion molecules in patients with acute coronary syndrome undergoing percutaneous coronary intervention. Results from the ARMYDA-ACS CAMs (Atorvastatin for Reduction of MYocardial Damage during Angioplasty-Cell Adhesion Molecules) substudy. *Journal of Cardiovascular Medicine*.

[58] Patti G., Chello M., Pasceri V. (2006). Protection from procedural myocardial injury by atorvastatin is associated with lower levels of adhesion molecules after percutaneous coronary intervention: results from the ARMYDA-CAMs (Atorvastatin for Reduction of MYocardial Damage during Angioplasty-Cell Adhesion Molecules) substudy. *Journal of the American College of Cardiology.*.

[59] Gottschalk O., Dao Trong M. L., Metz P. (2014). Simvastatin reduces leucocyte- and platelet-endothelial cell interaction in murine antigen-induced arthritis in vivo. *Scandinavian Journal of Rheumatology.*.

[60] Verdoia M., Nardin M., Negro F., Rolla R., De Luca G. (2018). Impact of statin therapy on the immature platelet count in patients with coronary artery disease: a single centre cohort study. *International Journal of Cardiology.*.

[61] Hasselbalch H. C., Riley C. H. (2006). Statins in the treatment of polycythaemia vera and allied disorders: an antithrombotic and cytoreductive potential?. *Leukemia Research.*.

[62] Nenna A., Lusini M., Spadaccio C. (2017). Preoperative atorvastatin reduces bleeding and blood products use in patients undergoing on-pump coronary artery bypass grafting. *Journal of Cardiovascular Medicine.*.

[63] Nenna A., Spadaccio C., Lusini M. (2019). Preoperative atorvastatin reduces bleeding and blood transfusions in patients undergoing elective isolated aortic valve replacement. *Interactive Cardiovascular and Thoracic Surgery*.

[64] Falcone G. J., Brouwers H. B., Biffi A. (2014). Warfarin and statins are associated with hematoma volume in primary infratentorial intracerebral hemorrhage. *Neurocritical Care.*.

[65] Quinn K. L., Macdonald E. M., Mamdani M. M., Diong C., Juurlink D. N. (2017). Lipophilic statins and the risk of intracranial hemorrhage following ischemic stroke: a population-based study. *Drug Safety*.

[66] Lin M. S., Lin Y. S., Chang S. T. (2019). Effect of initiating statin therapy on long-term outcomes of patients with dyslipidemia after intracerebral hemorrhage. *Atherosclerosis*.

[67] Atar S., Cannon C. P., Murphy S. A., Rosanio S., Uretsky B. F., Birnbaum Y. (2006). Statins are associated with lower risk of gastrointestinal bleeding in patients with unstable coronary syndromes: analysis of the Orbofiban in Patients with Unstable coronary Syndromes-Thrombolysis In Myocardial Infarction 16 (OPUS-TIMI 16) trial. *American Heart Journal*.

